# Alpha-Tocopherol Levels in Patients With Dentigerous Cysts: A Cross-Sectional Study

**DOI:** 10.7759/cureus.61359

**Published:** 2024-05-30

**Authors:** Nausathkhan Ubayathulla, Muthusekar M.R, Pratibha Ramani, Dharini S, Suvarna Kizhakkoottu, Karthikeyan Ramalingam

**Affiliations:** 1 Oral and Maxillofacial Surgery, Saveetha Dental College and Hospitals, Saveetha Institute of Medical and Technical Sciences, Saveetha University, Chennai, IND; 2 Oral and Maxillofacial Surgery, Emirates Health Services (EHS) Fujairah Specialized Dental Center and Hospital, Fujairah, ARE; 3 Oral Pathology and Microbiology, Saveetha Dental College and Hospitals, Saveetha Institute of Medical and Technical Sciences, Saveetha University, Chennai, IND

**Keywords:** alpha tocopherol, vitamin e, tocopherol, odontogenic cyst, dentigerous cyst

## Abstract

Background

Dentigerous cysts (DC) form due to fluid accumulation between the crown of the tooth and the reduced enamel epithelium. Due to the diverse clinical characteristics, such as ambiguity concerning their biological origins and the significance of timely diagnosis and detection of these lesions, researchers are presently motivated to undertake further investigations. The aim of the present study was to assess the amount of serum alpha-tocopherol in patients with DC and compare it with that of normal, healthy individuals.

Methods

A total sample size of n=34 was included in the current study. Group A, designated as the control group, comprised 17 randomly selected healthy subjects, while Group B, the DC diagnostic group, consisted of 17 patients. Blood samples were collected, and the concentration of vitamin E or alpha-tocopherol was evaluated and expressed in mg/mL.

Results

Compared to the mean vitamin E level in healthy controls (12.08 ± 1.92 mg/mL), patients with DC showed a statistically significant (p<0.0001) reduction in mean vitamin E levels (5.29 ± 1.01 mg/mL).

Conclusion

Patients with DC have lower levels of vitamin E than healthy individuals. The reduced concentration of vitamin E can have a role in the extension of cystic volume and thus have an impact on the aggressiveness of pathologic lesions. The therapeutic benefits of vitamin E supplementation in reducing the aggressiveness of DC should be evaluated in future research.

## Introduction

Dentigerous cysts (DC), which make up around 20% of oral cavity cysts, are among the most prevalent developmental odontogenic cysts, with a favorable prognosis and low recurrence. DC, a typical odontogenic cyst, is believed to originate from developmental factors and develop due to fluid accumulation between the crown of the tooth and the reduced enamel epithelium. It typically exhibits a non-recurrent nature and rarely recurs following excision [1,2]. The development of a dentigerous cyst can stop a tooth from erupting since it develops in relation to a tooth's crown, unlike the radicular cyst, which affects the tooth's roots. As a result, this cyst will appear radiographically as a well-defined radiolucent lesion surrounding the crown of an impacted tooth. The most frequently affected teeth are the third molars, followed by the canines. Enucleation of the cyst and extraction of the related tooth were required for the therapy of a dentigerous cyst.

The supporting stroma plays a vital role in maintaining epithelial tissues; however, slight modifications in the epithelium can trigger changes in the stroma, including the initiation of angiogenesis [1]. For the odontogenic epithelium to proliferate, it is crucial to have an adequate blood supply. Apoptosis may result from insufficient blood flow from the connective tissue because the epithelium lacks a vascular system. The tumor stroma unequivocally contains myofibroblasts, blood vessels, and inflammatory cells, all of which are crucial components in the growth and progression of tumors [3]. Neoplastic tissues require oxygen and essential nutrients for survival in order to keep up with their up-regulated growth potential. Because of this, neoplastic tissues stimulate angiogenesis, or neovascularization, at the time of tumor development [1,4].

A perfect angiogenesis indicator would be able to distinguish between newly formed blood vessels based on the quantity and quality of those vessels. The formation of new blood vessels from preexisting ones characterizes the complex physiological phenomenon known as angiogenesis. This process encompasses several phases, including the breakdown of the extracellular matrix, the proliferation and arrangement of endothelial cells, the formation of capillaries, and the establishment of connections between blood vessels [3]. Inflammatory reactions, tumors, wound healing, and tissue hypertrophy can result in neo-angiogenesis and an increase in blood supply to that particular area [2]. Previously reported data suggests that lesions could not grow larger than 1-2 mm in diameter without growing their network of capillary blood vessels [5]. Angiogenesis is controlled by various molecules like VEGF, CD31, CD34, von Willebrand factor, and CD105 (endoglin). Legan noticed that angiogenic endothelial cells exhibit distinct expression patterns of pan-endothelial markers (CD31, CD34, Factor VIII, and CD105) compared to endothelial cells in normal vessels [2,6].

Eight naturally occurring forms of vitamin E are: α-, β-, γ-, δ-tocopherol, and α-, β-, γ-, δ-tocotrienol [7,8]. Out of these eight, α-tocopherol is the main form found in human plasma [9,10]. Vitamin E possesses strong chain-breaking antioxidant properties, which are confined to lipid compartments like cell membranes because of its lipophilic nature. It stops lipids from oxidizing, protecting the integrity of cellular membranes [11]. Moreover, vitamin E is essential for erythrocyte stability as well as central and peripheral nerve conductivity [12,13]. The method for evaluating the α-tocopherol level is to measure the serum amounts of α-tocopherol. Peripheral neuropathy, spinocerebellar ataxia, and skeletal myopathy are identified as clear clinical indicators of vitamin deficiency when the serum levels of α-tocopherol drop below 8 mol/L [9]. The amount of α-tocopherol serum concentration required to prevent apparent (8 mol/L) or functional (12 mol/L) deficits in humans is not well understood. However, a growing body of research has shown that serum levels of α-tocopherol at or above 30 mol/L serum concentration have additional benefits, such as protection against several cancers and cardiovascular diseases [14,15]. 

Due to their possible anticancer effects, relatively non-toxic antioxidant nutrients such as beta-carotene, alpha-tocopherol, and glutathione, along with various retinoids, are currently the focus of extensive research. It has been proven that in addition to the regression of cancer, carotenoids, and tocopherols can inhibit and prevent the development of cancer. Many possible mechanisms, including programmed cell death, induction of cytotoxic cytokines, the regulation of gene expression, the blocking of the formation of the tumor's vital blood supply, or cellular differentiation, have been identified by recent research as ways in which these antioxidant nutrients inhibit the growth of cancer cells and kill them. Furthermore, research has validated the synergistic impacts of these antioxidants. They can cause cancer cells to die through several different processes, such as apoptosis, cytotoxic cytokine production, gene expression modification, blocking tumor angiogenesis, and encouraging cellular differentiation [16,17]. Of the varied forms of vitamin E, the only one that the body actively maintains is α-tocopherol. As a potent antioxidant, alpha-tocopherol works to combat free radicals and prevent the synthesis of nitrosamines, which are linked to the development of cancer [18,19].

Numerous studies in both humans and animals have found proof of the preventive impact of tocopherol supplementation in the prevention or treatment of chronic diseases, such as cancer and cardiovascular disorders [20,21]. In contrast to alpha-tocopherol, other variations of vitamin E demonstrate unique and superior biological characteristics. Recent mechanistic studies utilizing preclinical animal models suggest that these alternative forms may offer potential benefits for both the prevention and treatment of chronic diseases. A few long-chain vitamin E metabolites may also be more effective at reducing inflammation than their vitamin predecessors. The anti-inflammatory properties of these metabolites may be state-of-the-art, supporting the beneficial effects of vitamin E in vivo.

The effect of vitamin E on the development and pathogenesis of odontogenic cysts is less explored. It has previously been reported that the cystic lining can initiate endogenous prostaglandin production, which can induce the proliferation of epithelial cells, angiogenesis, and bone resorption, leading to cyst enlargement. In vitro studies have shown that vitamin E can downregulate phospholipase A2 and cyclooxygenase, thus resulting in a decreased production of prostaglandin E2. Since prostaglandin E2 is involved in cellular proliferation and angiogenesis, regulation of the prostaglandin feedback mechanism can be useful in down-regulating cellular proliferation and cyst enlargement [22].

According to our knowledge, no previous studies have been reported with context on the role of alpha-tocopherol levels in patients with dentigerous cysts. Since the development and enlargement of a cyst require increased proliferation activity of the epithelium and increased vascular supply, the established anti-tumorigenic functions of alpha-tocopherol can be studied in dentigerous cysts as well. Hence, the current study was done to evaluate the serum alpha-tocopherol (vitamin E) levels in patients diagnosed with dentigerous cysts and to evaluate the effect of vitamin E on the aggressiveness of dentigerous cysts. 

## Materials and methods

The sample population was chosen from the patients seeking treatment for oral conditions at the outpatient Department of Oral Medicine, Radiology, and Oral Surgery at Saveetha Dental College and Hospitals from 2019 to 2021 with an institutional human ethical committee number of SDC/PhD/07/18/47. The individuals were divided into two distinct groups, Group A and Group B, without considering age and gender. Group A was designated as the control group, comprising 17 healthy individuals randomly chosen, whereas Group B was identified as the study group, consisting of 17 diagnosed cases of dentigerous cysts with confirmed histopathological diagnosis. Patients with a previous history of tobacco chewing habits, vitamin supplementation, hypertension, diabetes mellitus, and other systemic diseases were excluded from both the case and control groups of the present study. 

Venipuncture was used for the case and control groups to obtain a 5 ml blood sample, which was then placed into a vial that had been previously sterilized without the use of ethylenediaminetetraacetic acid (EDTA). The samples were sent to a laboratory where the High-Performance Liquid Chromatography (HPLC) technique was used to analyze the serum levels of alpha-tocopherol. Known for its effectiveness, accuracy, and capacity to analyze complex mixtures quantitatively, this technique uses a series coupling of mass spectrometers. The apparatus is calibrated using tocopherol standards with individual concentrations ranging from 200 to 1800 ng. Hamilton syringes are then used to inject 50 μl of each standard into the reverse-phase high-performance liquid chromatography (RP-HPLC). After that, each standard is processed separately using the Isocratic program. The temperature is kept at 37°C, and the flow rate is set at 1.0 ml/min, with detection taking place at 280 nm. For every standard, a chromatogram will be produced. The two successive runs that show the same retention time will be chosen from among these, and their average will be computed for graph plotting in the calibration table. The process is called calibration, and the curve that is produced is called the calibration curve.

Centrifugation at 3000 rpm for 10 minutes is used to extract tocopherol from the sample after ethanol is used to precipitate the serum proteins. Before being injected into the HPLC, the organic solvent is first redissolved in ethanol and then evaporated in an atmosphere of nitrogen gas. A chromatogram is produced once the sample run is finished. For use in further calculations, the area of the peaks from the chromatogram is noted and calibrated in conjunction with the standards [23]. Alpha-tocopherol concentrations are expressed in milligrams per liter, or "mg/ml. An independent t-test was done to check the statistical significance with IBM SPSS Statistics for Windows, Version 23 (Released 2015; IBM Corp., Armonk, New York, United States).

## Results

In total, 34 samples were assessed in the present study. The total sample population consisted of 41.2% females and 58.8% males, respectively, with a mean age of 35.2 years. The control group comprised 6/17 females and 11/17 males, with a mean age of 39.4 years. Among the DC group samples, 8/17 were females and 9/17 cases were males, with a mean age of 30.7 years. Histopathology diagnosed 11/17 of the DC group as infected dentigerous cysts, while 6/17 remained non-infected. Table 1 depicts the age distribution of the selected cases and controls.

**Table 1:** Shows the distribution of the selected cases and controls according to the age groups DC: dentigerous cysts

Samples	<20 years	20-40 years	>40 years
Case (DC)	5	6	6
Control	0	9	8

**Figure 1:**
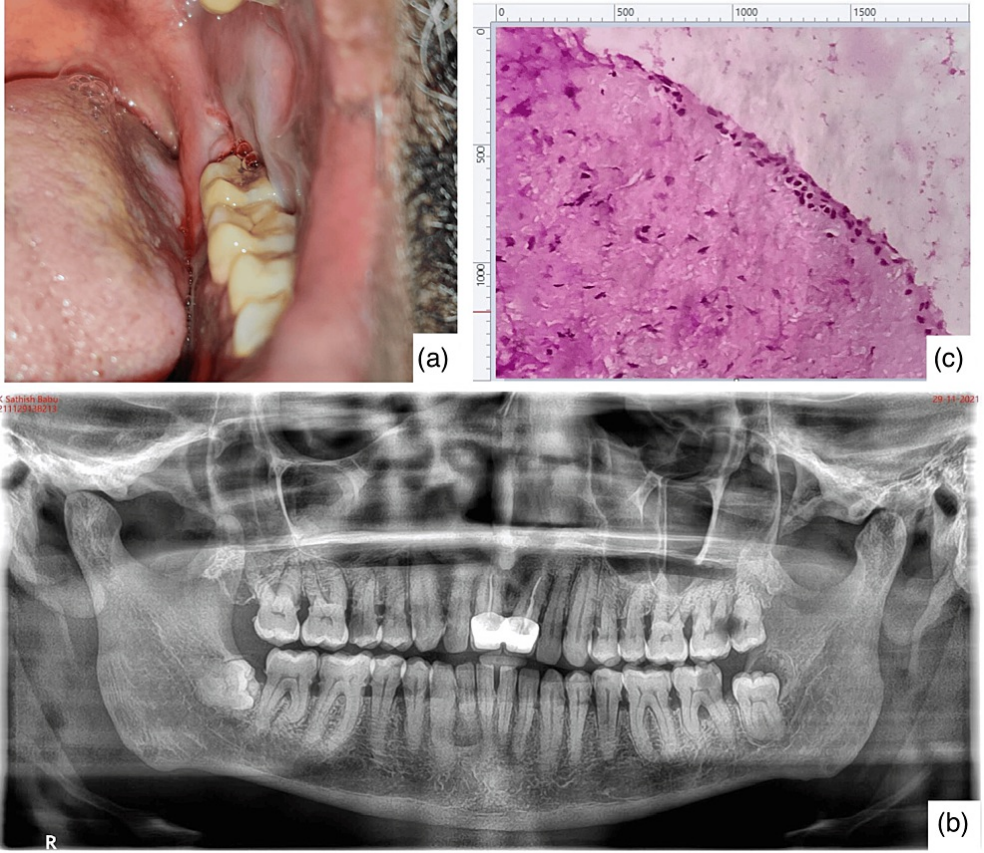
Shows images of a DC case in relation to 38 a: clinical picture; b: orthopantomograph shows a well-defined radiolucency in relation to the disto-occlusal crown portion of 38; c: photomicrograph of a hematoxylin and eosin-stained section shows a non-keratinized stratified squamous epithelium of 2-3 cell layer thickness along with a flat epithelial connective tissue interface (magnification 40X). DC: dentigerous cysts

The clinical picture, radiograph, and histopathological image of an included case are shown in Figure 1.

**Figure 2:**
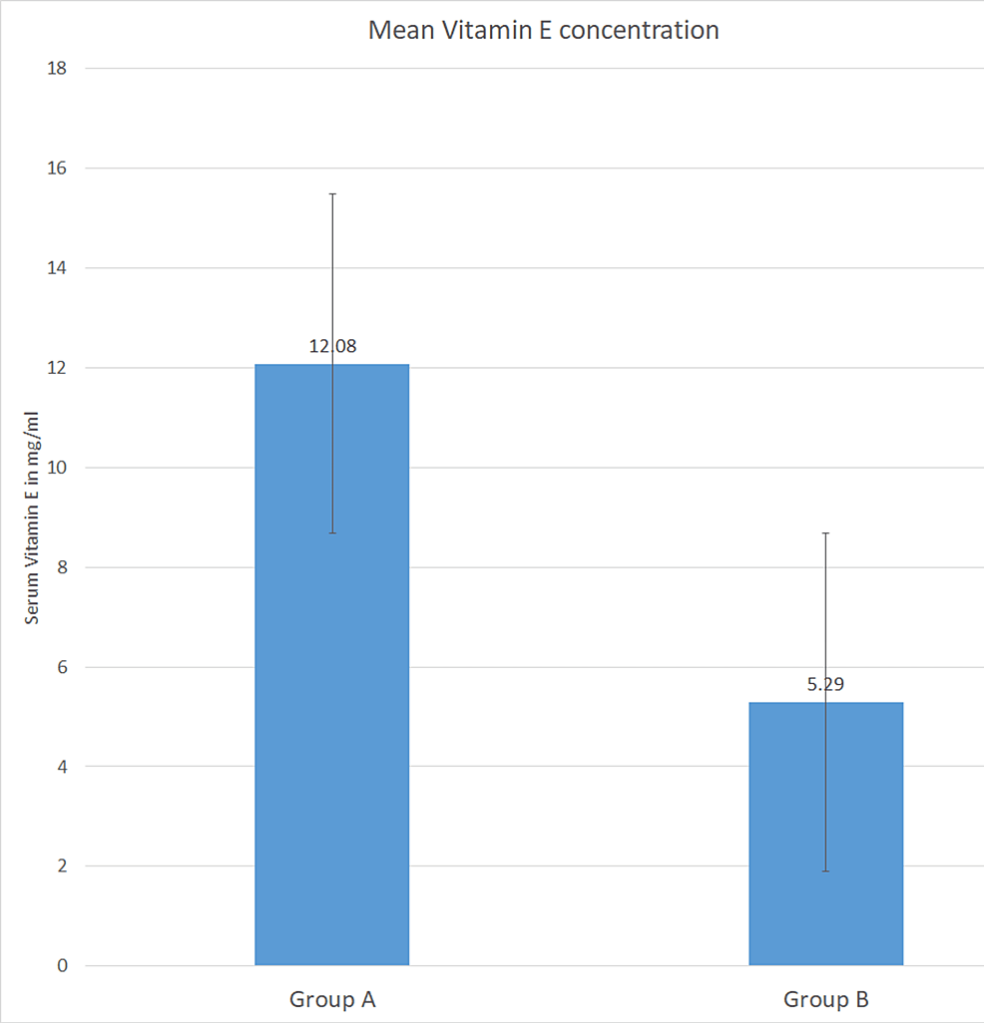
Mean values of serum vitamin E levels in mg/ml among Group A (control) and Group B (case) sample populations The error bars shown in the graph are standard errors.

The mean vitamin E levels of each age group in cases and controls were calculated. Among the cases, mean vitamin levels were 5.6 mg/mL (<20 years), 5.3 mg/mL (20-40 years), and 5.5 mg/mL (>40 years), respectively. In the control group, mean vitamin E levels were 12 mg/mL (20-40 years) and 11.7 mg/mL (>40 years). Vitamin E levels in both the case and control groups were subjected to an independent sample t-test. In comparison to healthy controls (mean ± SD = 12.08 ± 1.92 mg/mL), DC patients exhibited a lower mean vitamin E level (mean ± SD = 5.29 ± 1.01 mg/mL). The degree of freedom was 22, with a standard error of 0.738. Statistical analysis indicated a significant decrease in serum vitamin E levels among the DC group (P=0.0001) (Table 2 and Figure 2).

**Table 2:** Mean values of vitamin E levels in Group A and Group B

Group	N	Mean ± SD (mg/mL)	Df	P-value
Group A	17	12.08 ± 1.92	22	<0.0001
Group B	17	5.29 ± 1.01		

## Discussion

Vitamin E is receiving more attention for its potential to prevent precancerous lesions because of its antioxidant properties. It also acts as an essential nutrient that performs a variety of functions, such as scavenging free radicals, cytotoxic effects, preventing the growth and differentiation of cancer cells, preventing nitrosamine formation and mutagenicity, and hindering the synthesis of DNA and RNA in tumor cells [24]. 

Few animal studies have also proved the recovery of ovarian tissue from polycystic ovarian syndrome upon supplementation with vitamin E, and this indicated the therapeutic role of vitamin E in cystic lesions [25]. Studies correlating the effect of vitamin E on oral cystic lesions were sparse; however, few studies proposed that the serum vitamin E concentration was found to be significantly lower when compared to healthy controls in keratocystic odontogenic tumors and radicular cysts [26,27]. Our study is the first of its kind since we compared the serum concentration of vitamin E in DC and control groups. We observed that the mean alpha-tocopherol value of healthy controls was significantly high when compared to the mean vitamin E level of the DC group.

Serum vitamin E levels can vary according to age, diseases, and other systemic conditions. Since we selected the presence of any systemic conditions or disease as our exclusion criteria, the impact of those two factors was minimal in our study. However, we observed a slight reduction of mean serum vitamin E upon the advancement of age in both cases and controls. This can be due to the increased requirements during aging.

The primary reason for the development of DC in an impacted tooth is the pressure exerted on the impacted follicle, causing venous outflow obstruction and transudation of serum across the capillary walls, resulting in fluid accumulation between the crown and reduced enamel epithelium. It has already been proven that inflammation plays an important role in the enlargement of cysts. Despite their developmental origin, the majority of reported cases of DCs exhibit inflammation [28]. Studies have demonstrated that inflammatory events trigger the release of growth factors and cytokines, including interleukin 1, interleukin 6, and tumor necrosis factor. This will further lead to the induction of cellular stress and may initiate epithelial proliferation [29]. Compared to DC, inflammatory DC had noticeably higher basal and suprabasal layer cell PCNA expression, which explains the impact of inflammation on the proliferation of the cystic lining and enlargement of the cyst [30]. These cytokines and inflammatory mediators can also induce inflammatory mechanisms, which further result in the resorption of the peripheral bone. These epithelial proliferations and bone loss lead to the enlargement of the cyst. The complications of a long-standing DC are ameloblastoma, squamous cell carcinoma, and mucoepidermoid carcinoma. The inherent capacity of the cells of the cyst lining of DC to multiply in an uncoordinated manner correlates with its complications, such as ameloblastoma, squamous cell carcinoma, and mucoepidermoid carcinoma. It has been proven that vitamin E can inhibit cell proliferation and the activation of extracellular signal-regulated kinase in addition to its antioxidative action in the lung cancer model. It has been established that patients on hemodialysis supplemented with alpha or gamma-tocopherol showed a decrease in IL-6 and C-reactive protein levels, indicating a regressive effect of alpha-tocopherol on inflammation [29]. Hence, vitamin E can be considered an augmented therapeutic option in the treatment of dentigerous cysts [30].

Despite numerous clinical trials examining the potential benefits of other tocopherols, human studies investigating the positive effects of alpha-tocopherol are limited to a few clinical scenarios. Effects of other nutrients such as vitamin E, vitamin A, vitamin B12, lipoic acid, and biotin on epigenetics have been reviewed by Lod et al. and suggested that epigenetic changes can also have an impact on oral health and may influence both prognosis and treatment response [30]. Since alpha-tocopherol is capable of reducing pro-inflammatory mediators and cytokines, it can have an impact on the reduction of the inflammatory response and thereby the aggressiveness of DC. 

The present study carries limitations such as compromised geographical area coverage for sample selection, reduced sample population, and failure to take a complete diet history since alpha-tocopherol levels can vary according to the diet. 

Besides the above-mentioned limitations, the reduced mean vitamin E levels in DC compared to healthy controls in the present study can be an indicator pointing to the impact of vitamin E in the pathogenesis of DC. Hence, from a therapeutic point of view, the observations of the present study are valuable. In the future, large-scale prospective studies and randomized controlled trials can be conducted to check the therapeutic efficiency of vitamin E supplementation in reducing the aggressiveness of DC. Considering the actions of alpha-tocopherol on factors related to the etiopathogenesis of various odontogenic tumors and cysts, alpha-tocopherol might also have a possible role in contributing to the epigenetic modification needed for reducing the aggressiveness of DC.

## Conclusions

The present study showed that the mean vitamin E level in DC patients was significantly low when compared to healthy controls. This could be a sign of a possible in vivo role of vitamin E in the pathogenesis and progression of DC. Large-level studies can be conducted to evaluate the therapeutic efficiency of vitamin E supplementation in reducing the aggressiveness of DC among the affected patients. 
